# Focal impairment in myocardial fatty acid imaging in the left anterior descending artery area, a strong predictor for cardiac death in hemodialysis patients without obstructive coronary artery disease

**DOI:** 10.1007/s00259-015-3120-8

**Published:** 2015-06-27

**Authors:** Masato Nishimura, Tetsuya Hashimoto, Nagara Tamaki, Hiroyuki Kobayashi, Toshihiko Ono

**Affiliations:** Cardiovascular Division, Toujinkai Hospital, 83-1, Iga, Momoyama-cho, Fushimi-ku, Kyoto, 612-8026 Japan; Division of Urology, Toujinkai Hospital, Kyoto, Japan; Department of Nuclear Medicine, Hokkaido University Graduate School of Medicine, Sapporo, Japan

**Keywords:** Fatty acid imaging, Microcirculation, Myocardial infarction, Scintigraphy, Sudden death

## Abstract

**Purpose:**

We investigated whether impaired patterns of myocardial fatty acid imaging were associated with cardiac death in dialysis patients without coronary lesions.

**Methods:**

We prospectively enrolled 155 hemodialysis patients without obstructive coronary artery disease, who had been examined by single-photon emission computed tomography (SPECT) using the iodinated fatty acid analogue BMIPP. Uptake of BMIPP on SPECT was graded in 17 segments on a five-point scale (0, normal; 4, absent) and assessed as BMIPP summed scores. Of the enrolled 155 participants, we analyzed 95 who had BMIPP summed scores ≥ 6 (52 men and 43 women, 65 ± 11 years). BMIPP scores ≥ 2 in ≥ 2 consecutive segments in SPECT were defined as focal, and the others as non-focal pattern.

**Results:**

Of 95 participants analyzed, 42 (44.2 %) showed focal and 53 (55.8 %) non-focal type. During follow-up for 5.1 ± 2.0 years, 42 died of cardiac events. The occurrence of cardiac death was higher in the focal than in the non-focal group (30/42 [71.4 %] versus 12/53 [22.6 %], *p* = 0.001). In stepwise Cox hazard analysis, focal pattern was associated with cardiac death (hazard ratio 2.266), independent of impairment of BMIPP SPECT (BMIPP summed scores ≥ 12). The predictive potential of BMIPP SPECT for cardiac death was higher (*p* < 0.001) in the left anterior descending artery area compared with other coronary territories.

**Conclusions:**

Focal impairment in myocardial fatty acid imaging in the left anterior descending area may strongly predict cardiac death in this population.

## Introduction

Deaths of cardiac origin play an important role in the high mortality of patients with end-stage renal disease who undergo renal replacement therapy [1]. We have shown that visualizing impaired myocardial fatty acid metabolism by single-photon emission computed tomography (SPECT) using the iodinated fatty acid analogue, iodine-123-*β*-methyl iodophenyl-pentadecanoic acid (BMIPP), can help to identify patients at high risk of cardiac death among asymptomatic patients on hemodialysis who have not been treated by coronary intervention [2], and among those with coronary revascularization achieved by percutaneous coronary intervention [3]. This predictive potential of BMIPP SPECT for cardiac death in hemodialysis patients was identified in a multicenter cohort study in Japan (B-SAFE) [4]. Obstructive coronary artery disease (CAD) is undoubtedly involved in cardiac deaths induced by acute myocardial infarction (MI) or congestive heart failure (CHF) and in sudden cardiac death (SCD); however, cardiac deaths can occur in patients on hemodialysis without CAD [5]. Although myocardial fatty acid imaging reveals focal and non-focal impairment, it has not been elucidated whether either of these impaired pattern types of fatty acid imaging are involved in cardiac deaths of hemodialysis patients. In the present study, we investigated whether the impaired patterns of myocardial fatty acid imaging are associated with the cardiac deaths in hemodialysis patients without obstructive CAD, and further examined whether the predictive potential of impaired fatty acid imaging for cardiac deaths differed in the perfusion areas of the three coronary arteries.

## Material and methods

### Study population

**Fig. 1:**
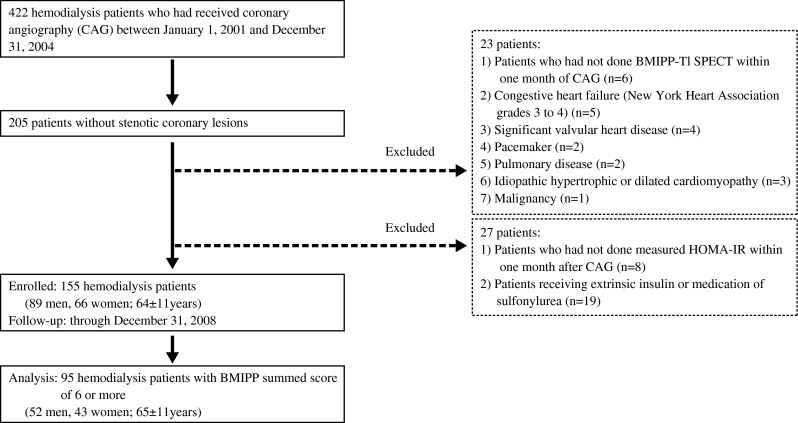
Entry and exclusion of study participants

This study was based on the data of a previously reported cohort study [6]. Among these data, 422 patients on maintenance hemodialysis at the Toujinkai group underwent coronary angiography (CAG) for the first time to determine the presence or absence of CAD between 1 January 2001 and 31 December 2004. The Toujinkai group includes Toujinkai Hospital, Toujinkai Clinic, and Toujinkai Satellite Clinic, Kyoto, Japan. No significant stenotic coronary lesions (Diameter stenosis greater than 50 %) were identified in 205 of the 422 patients. Among the 205, 50 patients were excluded from the study based on the reasons described in Fig. 1. Patients were not included in the study if they had a history of acute MI or invasive coronary artery intervention such as coronary artery bypass grafting or percutaneous coronary intervention, or unrecognized old MI without obstructive CAD identified as significantly reduced uptake on ^201^Thallium (Tl) SPECT images. Further, patients with CHF of NYHA grades III to IV, moderate or worse valvular heart disease, permanent pacemaker implantation or idiopathic cardiomyopathy were not included. Consequently, 155 hemodialysis patients without significant obstructive coronary lesions were enrolled in this cohort study. The participants were enrolled at the time of homeostatic model assessment of insulin resistance (HOMA-IR) measurements (between 1 January 2001 and 31 December 2004), and followed through 31 December 2008 until the endpoint described below was reached. Of the 155 subjects, 95 hemodialysis patients with abnormal BMIPP SPECT findings (BMIPP summed scores [SS] ≥ 6) were enrolled in the analysis (52 men, 43 women; age, 65 ± 11 years; mean dialysis duration, 87 ± 99 months); BMIPP SS ≥ 6 indicated the presence of myocardial ischemia in our previous study [7], and it was possible for us to distinguish between focal and non-focal types in the analysis of SPECT. Figure 1 summarizes entry and exclusion criteria for the study. The Ethics Committee for Human Research of Toujinkai Hospital approved this study, and all patients provided written informed consent for all procedures associated with the study prior to participation. The study was performed in accordance with the principles of the Declaration of Helsinki, and registered to the *ClinicalTrials.gov* (https://clinicaltrials.gov/): protocol identifier, NCT01068080.

### Radionuclide imaging

All patients underwent resting BMIPP and Tl dual myocardial scintigraphy after fasting for over 6 hours on a midweek, non-dialysis day within one month before the first CAG. Patients were injected at rest intravenously with 111-MBq of ^123^I-BMIPP (Nihon Medi-Physics, Tokyo, Japan) and 111 MBq of ^201^Tl (Nihon Medi-Physics). Details of the dual BMIPP-Tl SPECT procedure are described elsewhere [2–4, 6, 7]. The images of the left ventricle were divided into 17 segments for semiquantitative analysis, and coronary perfusion territories of these 17 segments were determined according to the standard myocardial segmentation for tomographic heart imaging established by the American Heart Association [8]. The amount of radioactivity taken up by each segment was visually graded and assigned an uptake score of 0 (normal), 1 (mildly reduced), 2 (moderately reduced), 3 (severely reduced), or 4 (none). The BMIPP and Tl SPECT scores for 17 myocardial segments were designated as summed BMIPP and Tl scores, respectively. A perfusion metabolic mismatch score between BMIPP and Tl in a whole SPECT was obtained as BMIPP SS minus Tl SS, and BMIPP-Tl mismatch score in each coronary territory was obtained as total BMIPP score minus total Tl score in that territory. The same experienced technician performed all scintigraphic procedures. All BMIPP and Tl SPECT images were interpreted within one week of the SPECT examination by the same two investigators who were blinded to clinical and laboratory information about the patients. The inter-observer and intra-observer variability in the BMIPP SS at our institute was 6.8 ± 1.4 % and 5.4 ± 1.4 %, respectively. Patients were divided into two groups according to the myocardial imaging patterns in BMIPP SPECT. The focal pattern was defined as BMIPP defect scores ≥ 2 in ≥ 2 consecutive left ventricular segments. Minimally impaired uptake (BMIPP score ≤ 1) or multiple small defects with the size of each defect ≤ 1 segment but BMIPP scores ≥ 2 were defined as non-focal.

### Echocardiography

The patients underwent two-dimensionally guided echocardiography using a single ultrasonographic recorder (UF-8800, Fukuda Denshi, Tokyo, Japan) on a midweek non-dialysis day within one month before CAG. Left ventricular dimensions and left ventricular ejection fraction (LVEF) were quantified using the biplanar Simpson’s rule, and left ventricular mass was measured as recommended by the American Society of Echocardiography [9]. Left ventricular mass was normalized to body surface area, and is described herein as left ventricular mass index.

### Biochemical and hematological determinations

On a midweek dialysis day within 30 days after CAG, blood samples (10 ml) were obtained in the morning from patients who had fasted overnight and rested for 10 min. Blood hemoglobin, plasma concentrations of intact parathyroid hormone and B-type natriuretic peptide, and serum concentrations of calcium, inorganic phosphorus, albumin, total cholesterol, and C-reactive protein were determined.

### Assessment of insulin resistance

We used fasting plasma glucose and fasting plasma insulin concentrations to calculate the HOMA-IR: fasting glucose concentration (mmol/L) × fasting insulin concentration (μU/ml)/22.5. Blood samples were collected on the same day to measure other biochemical and hematological parameters.

### Endpoint

The endpoint was cardiac-derived death, that is, SCD or death due to acute MI or CHF. We defined SCD as death within 24 hours of the time that the patient was last seen alive in a normal state of health and for which a cardiac disease such as malignant arrhythmia or acute coronary syndrome was considered the cause. Cerebrovascular accidents were ruled out by post-mortem examinations. Acute MI was diagnosed when new abnormal Q waves appeared on electrocardiogram together with anterior chest pain or discomfort, when abnormal left ventricular wall motion was recognized on echocardiogram, and when serum concentrations of troponin-T and creatine phosphokinase-MB fraction were significantly elevated. Death due to CHF was diagnosed when the patients had died of primary heart failure of cardiac origin; the patients who had died of secondary heart failure following acute MI or who died in a state of chronic excess fluid accompanied with non-cardiac diseases were not included. Blinded investigators within the Toujinkai group or Kyoto Second Red Cross Hospital adjudicated all endpoints.

### Statistical analysis

Values are expressed as means ± SD. We compared the means of continuous variables using non-paired *t* tests. Categorical data were analyzed using the χ^2^ test. Prognosis was assessed using univariate and multivariate Cox proportional hazards models. Covariates that were significant in univariate Cox hazard analyses (*p* < 0.05) were assessed by stepwise Cox hazard analyses. We examined event-free survival using the Kaplan-Meier method and the log-rank test. *p* values of < 0.05 were considered significant. The predictive potential for cardiac death was compared among the total BMIPP scores in the coronary artery territories using receiver operating characteristic (ROC) analysis. All data were statistically analyzed by individuals who were blinded to any clinical information about the patients. All statistical analyses were performed with SAS software version 8.2.

## Results

**Fig. 2:**
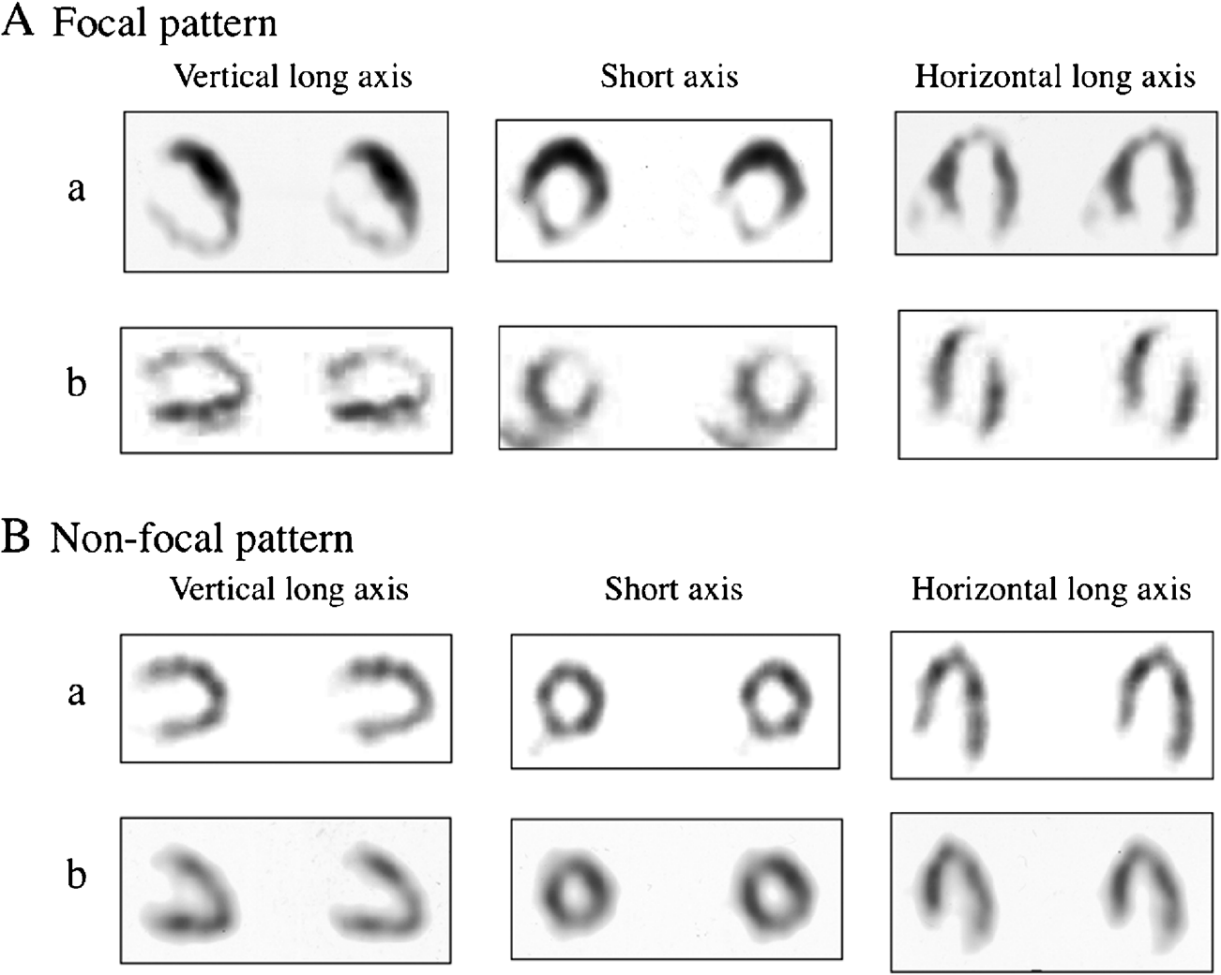
BMIPP SPECT images of focal (A) and non-focal (B) patterns. **A** Focal pattern of BMIPP SPECT. a BMIPP summed score 20 (right coronary artery area 15, left anterior descending artery area 3, left circumflex artery area 2), b BMIPP summed score 30 (right coronary artery area 6, left anterior descending artery area 18, left circumflex artery area 6); **B** Non-focal pattern of BMIPP SPECT. a BMIPP summed score 8 (right coronary artery area 4, left anterior descending artery area 3, left circumflex artery area 1), b BMIPP summed score 17 (right coronary artery area 6, left anterior descending artery area 6, left circumflex artery area 5)

Of the 95 enrolled patients analyzed, focal pattern in BMIPP SPECT was recognized in 42 patients (44.2 %) and non-focal pattern in 53 patients (55.8 %). Figure 2 shows representative cases of focal and non-focal pattern in BMIPP SPECT. Table 1 shows baseline clinical characteristics in the focal and non-focal groups. The mean BMIPP SS was higher in the focal than in the non-focal group, whereas the mean Tl SS did not differ between the two groups. The percentages of BMIPP SS ≥ 12, a cutoff value of predicting cardiac death in our previous studies [2, 6], were higher in the focal than in the non-focal group.

**Table 1:** Differences in clinical characteristics of focal and non-focal groups

	Focal (*n* = 42)	Non-focal (*n* = 53)	*p*
Age, years	68.2 ± 10.7	62.8 ± 10.4	*0.022*
Male gender, n (%)	29 (69.0)	23 (43.4)	*0.013*
Dialysis duration, months	83.8 ± 95.3	88.8 ± 103.1	*0.651*
Smoking habit, n (%)	17 (40.5)	17 (32.1)	*0.396*
Alcohol consumption, n (%)	17 (40.5)	16 (30.2)	*0.296*
Diabetes mellitus, n (%)	17 (40.5)	19 (35.8)	*0.644*
Systolic blood pressure before dialysis, mm Hg	143.2 ± 13.0	144.9 ± 17.1	*0.527*
Diastolic blood pressure before dialysis, mm Hg	77.7 ± 21.9	81.5 ± 20.0	*0.583*
Body mass index, kg/m^2^	20.7 ± 4.2	21.6 ± 4.3	*0.596*
Cardiothoracic ratio, %	52.6 ± 6.0	53.0 ± 4.7	*0.731*
Left ventricular ejection fraction, %	57.1 ± 17.9	63.2 ± 13.1	*0.112*
Left ventricular mass index, g/m^2^	142.5 ± 45.3	144.9 ± 17.1	*0.527*
Blood hemoglobin, g/L	103.2 ± 8.8	104.0 ± 12.1	*0.860*
Serum albumin, g/L	37.8 ± 3.2	37.8 ± 3.8	*0.885*
Serum calcium, mmol/L	2.3 ± 0.2	2.3 ± 0.2	*0.835*
Serum inorganic phosphorus, mmol/L	1.6 ± 0.4	1.7 ± 0.3	*0.372*
Serum total cholesterol, mmol/L	4.3 ± 0.8	4.5 ± 0.9	*0.627*
Log serum intact parathyroid hormone, ng/L	2.3 ± 0.4	2.2 ± 0.3	*0.492*
Serum C-reactive protein, mg/L	4.2 ± 3.4	3.8 ± 2.7	*0.697*
Log plasma B-type natriuretic peptide, pg/ml	2.4 ± 0.5	2.3 ± 0.4	*0.882*
HOMA-IR, mmol/L · μU/ml	5.7 ± 2.0	5.4 ± 2.0	*0.446*
BMIPP summed scores	17.4 ± 9.0	12.0 ± 5.2	*<0.001*
BMIPP summed scores ≧12, n (%)	30 (71.4)	26 (49.1)	*0.028*
Tl summed scores	6.3 ± 4.1	6.7 ± 4.7	*0.694*
Medications			
*α*_1_ blockers, n (%)	4 (9.5)	5 (9.5)	*1.000*
*β* blockers, n (%)	13 (31.0)	16 (30.2)	*0.936*
Calcium channel blockers, n (%)	15 (35.7)	15 (28.3)	*0.644*
ACEI, n (%)	0 (0)	2 (3.8)	*0.502*
ARB, n (%)	11 (26.2)	16 (30.2)	*0.668*
Nitrates, n (%)	3 (7.1)	9 (17.0)	*0.152*
Antiplatelet drugs, n (%)	24 (57.1)	26 (49.1)	*0.433*
Anticoagulation drugs, n (%)	1 (2.4)	0 (0)	*0.442*
Statins, n (%)	9 (21.4)	14 (26.4)	*0.573*
Vitamin D, n (%)	25 (59.5)	27 (50.9)	*0.404*

*HOMA-IR* the homeostasis model assessment index of insulin resistance, *ACEI* angiotensin I converting enzyme inhibitors, *ARB* angiotensin II type-1 receptor antagonists

### Cardiac deaths and fatty acid imaging patterns

Of the 95 enrolled patients, 42 (44.2 %) died of cardiac events (acute MI, *n* = 7; CHF, *n* = 18; SCD, *n* = 17) and 14 (14.7 %) died of non-cardiac causes (malignancy, *n* = 5; infectious diseases, *n* = 3; cerebrovascular accidents, *n* = 3; respiratory diseases, *n* = 1; hepatic diseases, *n* = 1; gastrointestinal bleeding, *n* = 1) during follow-up. The incidences of cardiac deaths were higher in the focal than in the non-focal group (30/42, 71.4 % versus 12/53, 22.6 %; *p* <0.001). Compared with the non-focal group, the focal group showed higher incidence of all cause deaths and cardiac deaths induced by acute MI or SCD; however, the incidence of CHF death did not differ between focal and non-focal groups (Table 2). In univariate Cox hazard analysis for cardiac deaths, age, HOMA-IR, BMIPP SS, BMIPP SS ≥ 12, and focal pattern in BMIPP SPECT were positively, and body mass index and serum albumin concentration were inversely associated with cardiac deaths (Table 3). In stepwise Cox hazard analysis for cardiac deaths among age, body mass index, serum albumin, HOMA-IR, BMIPP SS ≥ 12, or focal pattern in BMIPP SPECT, focal pattern in BMIPP SPECT was associated with cardiac deaths independently of BMIPP SS ≥ 12 (Table 4).

**Table 2:** All-cause and cardiac deaths

	Focal (*n* = 42)	Non-focal (*n* = 53)	*p*
All-cause deaths	34 (81.0)	22 (41.5)	*< 0.001*
Cardiac deaths	30 (71.4)	12 (22.6)	*< 0.001*
Acute myocardial infarction death	7 (16.7)	0 (0)	*0.002*
Heart failure death	9 (21.4)	9 (17.0)	*0.583*
Sudden cardiac death	14 (33.3)	3 (5.7)	*<0.001*

**Table 3:** Univariate Cox hazard analysis for cardiac death

	Hazard ratio	95 % CI	*p*
Age (1 year)	1.049	1.019-1.080	*0.001*
Male gender (0 = female; 1 = male)	0.990	0.539-1.820	*0.975*
Dialysis duration (1 month)	1.002	0.999-1.005	*0.261*
Smoking habit (0 = no; 1 = yes)	0.904	0.475-1.718	*0.757*
Alcohol consumption (0 = no; 1 = yes)	0.713	0.364-1.398	*0.325*
Diabetes mellitus (0 = no; 1 = yes)	0.981	0.522-1.846	*0.953*
Systolic blood pressure before dialysis (1 mmHg)	0.990	0.971-1.009	*0.292*
Diastolic blood pressure before dialysis (1 mmHg)	0.991	0.973-1.009	*0.322*
Body mass index (1 kg/m^2^)	0.903	0.839-0.971	*0.006*
Cardiothoracic ratio (1 %)	1.029	0.972-1.089	*0.331*
Left ventricular ejection fraction (1 %)	0.994	0.976-1.013	*0.522*
Left ventricular mass index (1 g/m^2^)	0.996	0.989-1.003	*0.247*
Blood hemoglobin (1 g/L)	0.990	0.963-1.018	*0.497*
Serum albumin (1 g/L)	0.891	0.818-0.970	*0.008*
Serum calcium (1 mmol/L)	0.545	0.072-4.103	*0.555*
Serum inorganic phosphorus (1 mmol/L)	0.487	0.209-1.134	*0.095*
Serum total cholesterol (1 mmol/L)	0.777	0.546-1.108	*0.163*
Log serum intact parathyroid hormone (1)	1.044	0.424-2.571	*0.925*
Serum C-reactive protein (1 mg/L)	0.923	0.827-1.030	*0.151*
Log plasma B-type natriuretic peptide (1)	0.660	0.307-1.418	*0.287*
HOMA-IR (1 mmol/L · μU/ml)	1.293	1.123-1.488	*< 0.001*
BMIPP summed scores (1)	1.057	1.026-1.089	*< 0.001*
BMIPP summed scores ≥ 12 (0 = no; 1 = yes)	13.368	4.122-43.361	*< 0.001*
Tl summed scores (1)	1.054	0.988-1.123	*0.110*
Focal pattern in BMIPP SPECT (0 = no; 1 = yes)	3.814	1.947-7.471	*< 0.001*
Medications			
*α*_1_ blockers (0 = no; 1 = yes)	0.929	0.331-2.609	*0.889*
*β* blockers (0 = no; 1 = yes)	1.190	0.624-2.272	*0.597*
Calcium channel blockers (0 = no; 1 = yes)	0.903	0.468-1.739	*0.760*
ACEI (0 = no; 1 = yes)	1.827	0.248-13.343	*0.554*
ARB (0 = no; 1 = yes)	1.162	0.593-2.279	*0.662*
Nitrates (0 = no; 1 = yes)	0.804	0.316-2.046	*0.647*
Antiplatelet drugs (0 = no; 1 = yes)	1.091	0.591-2.013	*0.780*
Anticoagulation drugs (0 = no; 1 = yes)	1.450	0.199-10.595	*0.714*
Statins (0 = no; 1 = yes)	0.490	0.206-1.165	*0.107*
Vitamin D (0 = no; 1 = yes)	0.697	0.380-1.278	*0.234*

*CI* confidence interval; *HOMA-IR* the homeostasis model assessment index of insulin resistance; *ACEI* angiotensin I converting enzyme inhibitors; *ARB* angiotensin II type-1 receptor antagonist

**Table 4:** Stepwise Cox hazard analysis for cardiac death

	Hazard ratio	95 % CI	*p*
Focal pattern in BMIPP SPECT (0 = no; 1 = yes)	2.258	1.136-4.486	*0.020*
BMIPP summed scores ≥12 (0 = no; 1 = yes)	8.712	2.546-29.808	*0.001*
Serum albumin (1 g/L)	0.882	0.792-0.981	*0.021*
HOMA-IR (1 mmol/L · μU/ml)	1.150	0.985-1.342	*0.077*
Body mass index (1 kg/m^2^)	0.940	0.877-1.008	*0.082*

*CI* confidence interval; *HOMA-IR* the homeostasis model assessment index of insulin resistance

**Fig. 3:**
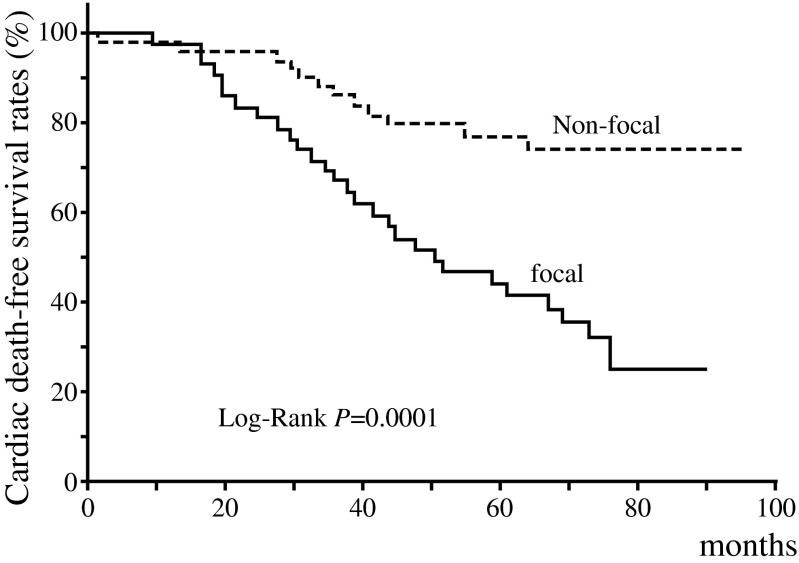
Kaplan-Meier analysis of cardiac death-free survival of focal (*n* = 42) and non-focal (*n* = 53) groups

**Fig. 4:**
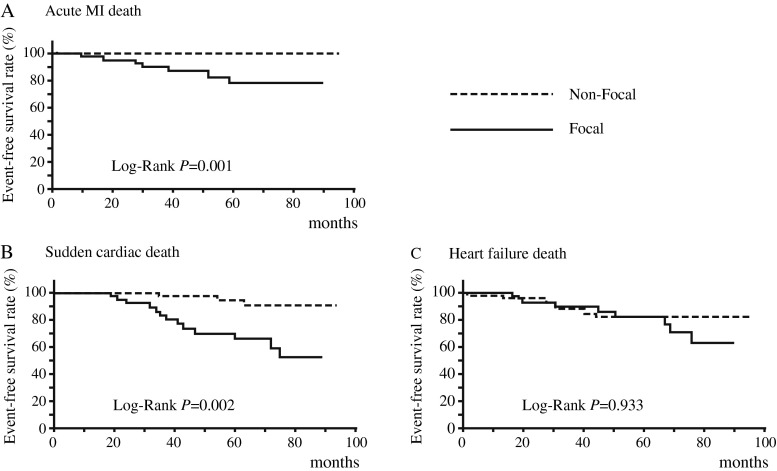
Kaplan-Meier analyses of cardiac death-free survival in focal and non-focal groups by types of cardiac deaths. *MI,* myocardial infarction

In Kaplan-Meier survival analysis, cardiac death-free survival rates for 5 years were lower in the focal than in the non-focal group (43.7 % versus 77.2 %, *p* = 0.001) (Fig. 3). Among the types of cardiac deaths, the focal group showed lower event-free survival rates than the non-focal group in acute MI death (5 years: 77.0 % versus 100 %, *p* = 0.001) or SCD (5 years: 69.8 % versus 94.4 %, *P* = 0.002); however, event-free survival rate of CHF death did not differ between the focal and non-focal groups (5 years: 81.3 % versus 81.7 %, *p* = 0.933) (Fig. 4).

### Impaired fatty acid imaging and cardiac deaths in the coronary artery territories

**Fig. 5:**
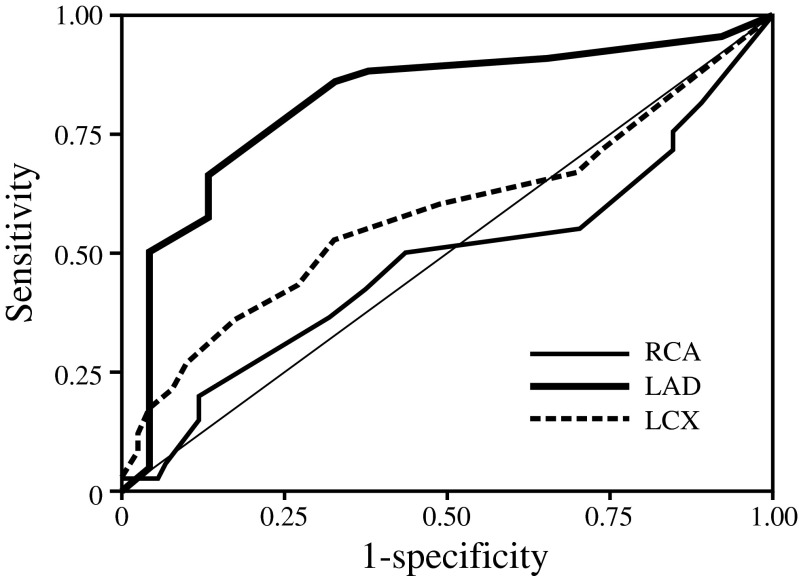
Receiver operating characteristic analysis regarding the predictive potential for cardiac death by coronary territorial impairment in BMIPP SPECT. The area under the curve: LAD, 0.818; LCX, 0.582; RCA, 0.478. *RCA,* right coronary artery; *LAD,* left anterior descending artery; *LCX,* left circumflex artery

Among 42 patients of the focal group, 20 patients showed focally reduced uptake of BMIPP in SPECT in the single coronary artery area; right coronary artery (RCA), *n* = 7; left anterior descending artery (LAD), *n* = 6; left circumflex artery (LCX), *n* = 7. The other 22 patients showed focally reduced uptake of BMIPP in the multiple coronary artery areas; RCA + LAD, *n* = 9; LAD + LCX, *n* = 8; RCA + LCX, *n* = 2; RCA + LAD + LCX, *n* = 3. Consequently, focally reduced uptake of BMIPP in the RCA, LAD, or LCX area was recognized in 21/42 (50.0 %), 26/42 (61.9 %), or 20/42 (47.6 %), respectively. In univariate Cox hazard analysis, focally reduced uptake of BMIPP in SPECT was significantly associated with cardiac death in the LAD area (hazard ratio, 3.152; 95 % CI, 1.276-7.786, *p* = 0.013), but not in the RCA (hazard ratio, 1.086; 95 % CI, 0.524-2.253, *p* = 0.824) or LCX area (risk ratio, 0.726; 95 % CI, 0.346-1.525; *p* = 0.398). The mean total BMIPP scores in the coronary perfusion areas of the focal group were higher than those of non-focal group in the LAD area (10.5 ± 6.4 versus 4.3 ± 2.1, *p* <0.001), but not in either the RCA (3.5 ± 3.6 versus 4.0 ± 2.2, *p* = 0.676) or LCX area (3.4 ± 4.5 versus 3.6 ± 2.7, *p* = 0.956). In univariate Cox hazard analysis, total BMIPP scores in the coronary territories were significantly associated with cardiac death in the LAD area (hazard ratio, 1.092; 95 % CI, 1.047-1,139; *p* < 0.001), but not in the RCA (hazard ratio, 0.998; 95 % CI, 0.895-1.114; *p* = 0.977) or LCX area (hazard ratio, 1.069; 95 % CI, 0.991-1.153; *p* = 0.083). In the ROC analysis, the predictive potential of coronary territorial BMIPP scores for cardiac deaths was higher (*p* < 0.001) in the LAD area than the LCX or RCA areas (Fig. 5).

### Perfusion metabolic mismatch and cardiac death

Of 95 patients, BMIPP SS was higher than Tl SS in 94. One patient showed lower BMIPP SS than Tl SS; total BMIPP scores were lower than total Tl scores in the LAD (three versus four) and RCA (two versus eight) areas. No other patients showed higher Tl score than BMIPP score in any coronary territorial area. The predictive potential of BMIPP-Tl mismatch score for cardiac death was recognized in a whole SPECT (hazard ratio, 1.044; 95 % CI, 1.013-1.075; *p* = 0.005), and was found in the LAD area (hazard ratio, 1.075; 95 % CI, 1.030-1.122; *p* = 0.001), but not in the LCX or RCA areas. However, in comparison between BMIPP score and BMIPP-Tl mismatch score using Cox hazard analysis, BMIPP SS (hazard ratio, 1.080; 95 % CI, 1.013-1.152, *p* = 0.018) and total BMIPP score in the LAD (hazard ratio, 1.185; 95 % CI, 1.046-1.341; *p* = 0.007) were more useful for prediction of cardiac death than BMIPP-Tl mismatch score in a whole SPECT (hazard ratio, 0.977; 95 % CI0.918-1.039; *p* = 0.453) and that in the LAD area (hazard ratio, 0.916; 95 % CI 0.808-1.038; *p* = 0.170), respectively.

### Impaired fatty acid imaging and left ventricular systolic function

BMIPP SS tended to be inversely related with LVEF (*r* = -0.181, *p* = 0.079), whereas Tl SS was not related with LVEF (*r* = 0.050, *p* = 0.632). Further, LVEF tended to be inversely associated with total BMIPP scores in the LAD area (*r* = -0.201, *p* = 0.051), but not in the LCX (*r* = -0.055, *p* = 0.599) or RCA (*r* = -0.030, *p* = 0.776) areas.

## Discussion

Of 95 patients who had BMIPP SS of six or more, focal pattern was recognized in 42 (44.2 %), whereas non-focal pattern was recognized in 53 (55.8 %). During follow up for 5.1 ± 2.0 years, 42 patients died of cardiac events. The occurrence of cardiac deaths was higher in the focal than in the non-focal group. In stepwise Cox hazard analysis, focal pattern was associated with cardiac death independently of BMIPP SS ≥ 12, a cutoff value predicting cardiac death in our previous studies [2, 6]. Cardiac death-free survival rates at 5 years were lower in the focal than in the non-focal types. The mean total BMIPP scores of the coronary territories were higher in the focal than in the non-focal group in the LAD area, but not in the RCA or LCX areas, and the predictive potential of reduced uptake of BMIPP was stronger in the LAD area compared with the RCA or LCX areas. These findings indicate that not only severe impairment in BMIPP SPECT, but also focally impaired myocardial fatty acid imaging in the LAD area, could be strong risk factors for cardiac death in this population.

^123^I-BMIPP is a branched free fatty acid (FFA) analogue characterized by resistance to β-oxidation. Intravenously administered BMIPP is incorporated from the blood into myocardial cells via the CD36-positive FFA binding protein. Incorporated BMIPP is acylated by resolution of adenosine triphosphate (ATP). Acylated BMIPP is mostly accumulated in the lipid pool, and a part of acylated BMIPP is metabolized to ρ-iodophenyl acetic acid via α-oxidation or β-oxidation in mitochondria. Of intracoronary administered BMIPP in canine myocardium, uptake into myocardial cells was 74 %, and retention of acylated BMIPP in the lipid pool was 65.3 %, whereas metabolism via α-oxidation or β-oxidation was only 8.7 %. BMIPP SPECT imaging is believed to reflect intracardiac accumulation of acylated BMIPP in the lipid pool and myocardial content of ATP [10–15].

Impaired fatty acid metabolism and consequent accumulation of acyl CoA are characteristic of renal failure [16]. Accumulated acyl CoAs inhibit glucose uptake by disruption of the intracellular signaling cascade that moves the GLUT4 transporter from its intracellular location to the surface of the myocardial membrane [17, 18], and also inhibit enzymes important in glucose metabolism such as pyruvate dehydrogenase [19, 20]; accumulated acyl CoAs thereby enhance insulin resistance. In our previous study, impaired fatty acid metabolism evaluated by BMIPP SPECT was in proportion to HOMA-IR, an indicator of insulin resistance, in diabetic and nondiabetic hemodialysis patients without obstructive CAD [21]. Therefore, impairment in BMIPP SPECT in hemodialysis patients without obstructive CAD may indicate the reduction of myocardial ATP synthesis via inhibition of not only fatty acid but also glucose metabolism.

Mechanical stresses to the heart by continued pressure and/or volume overloading and reduction of myocardial blood supply are thought to be the main factors impairing myocardial fatty acid metabolism in hemodialysis patients. Since the former is inevitable in patients on maintenance hemodialysis, myocardial fatty acid metabolism has a tendency to be impaired in this population. In pathological studies, intramyocardial arteriolar thickening, reduced capillary density, and myocardial fibrosis are unique findings of the heart of hemodialysis patients [22–24]; these myocardial abnormalities can potentially cause myocardial microcirculatory disturbance. These characteristics in hemodialysis patients give rise to the result that the myocardial cells are more susceptible to reduced myocardial blood supply than in non-dialysis patients, and lead to the impairment in myocardial fatty acid metabolism. When significant obstructive coronary lesions are not found, as in this study population, myocardial microcirculation disorder may be the main factor causing significant impairment of myocardial fatty acid metabolism.

In the present study, reduction of BMIPP in SPECT in the LAD area strongly predicted cardiac death, as compared with the areas of LCX or RCA. Since the LAD perfuses important areas of the left ventricle as the pump function of the heart, reduction in ATP synthesis in this perfusion area shown by impaired BMIPP SPECT might result in severe cardiac events leading to cardiac deaths. What is the mechanism of focal impairment in myocardial fatty acid metabolism? The relation between endothelial dysfunction in the epicardial coronary arteries and microcirculation disturbance in the coronary perfusion areas might partly explain the mechanism. Coronary endothelial dysfunction is detected in some patients with minimally obstructive CAD [25]. Endothelium-dependent vasodilation was less evident in a group with than without reversible defects on myocardial perfusion images among patients without significant obstructive CAD [26]. Prior studies have demonstrated that coronary microvascular endothelial dysfunction may contribute to the reduced vasodilator reserve in patients with minimally obstructive CAD [27, 28]. Hasdai et al. reported that coronary endothelial dysfunction in humans may cause dysfunction of microcirculation endothelium of the coronary territory, which results in reduction of myocardial blood flow [29]. Further, endothelial function was impaired in isolated human uremic resistance arteries [30]. Endothelial dysfunction in the epicardial coronary arteries might have caused myocardial microcirculation disturbance via microcirculation endothelial dysfunction or spasm [31], leading to impairment in myocardial fatty acid metabolism in the coronary perfusion areas.

Slim et al. reported that location of defect on exercise or pharmacological stress myocardial perfusion imaging was not associated with future hard cardiac events such as cardiac death or non-fatal myocardial infarction in patients with known or suspected coronary artery disease [32]. The difference in the results obtained by Slim et al. and ourselves might have originated from the difference between myocardial perfusion imaging and fatty acid imaging, and that between the patient populations (non-dialysis and dialysis patients). Matsuki et al. previously reported that the predictive potential for the hard cardiac events was higher in BMIPP SPECT than stress myocardial perfusion SPECT, and BMIPP SPECT imaging had excellent negative predictive values in patients with angina [33]. Because BMIPP SPECT is believed to reflect myocardial ATP synthesis [10–15], regional reduction in myocardial energy supply in an important coronary territory area such as LAD might be associated with cardiac prognosis particularly in hemodialysis patients, although stress myocardial perfusion imaging is also useful for risk stratification of cardiovascular events in this population [34, 35]. In the present study, BMIPP SS tended to be inversely related with LVEF, whereas Tl SS was not related with LVEF. Furthermore, LVEF tended to be inversely associated with total BMIPP scores in the LAD area, but not in the LCX or RCA areas. Since LVEF is one of the major predictors for cardiovascular events in asymptomatic hemodialysis patients [36], this relation between impaired BMIPP uptake and left ventricular systolic dysfunction may contribute to explaining in part how impaired fatty acid imaging in the LAD area could be associated with cardiac death. Further investigation is needed to clarify this important issue.

This study has several limitations. We used CAG to confirm the presence of suspected myocardial ischemia in the patients. The inclusion criterion of “without significant obstructive coronary artery disease” does not necessarily mean that the epicardial coronary arteries were normal. Since histopathological and intravascular ultrasound studies have demonstrated the propensity of angiography to underestimate lesional severity [37, 38], we cannot exclude the possibility that some angiographically non-significant lesions were flow limiting. We used HOMA-IR to evaluate levels of insulin resistance. Although others have shown that HOMA-IR closely correlates with clamp-measured insulin resistance in patients with or without diabetes mellitus, including those with chronic renal failure [39], the use of HOMA-IR can be considered as a limitation of this study. We did not investigate the relation between regional wall motion of the left ventricle and uptake of BMIPP and/or Tl in SPECT. Since this study was a retrospective analysis of a previous cohort study [6], we could not define the predictive value of focal impairment of BMIPP SPECT for cardiac deaths.

The results of this study showed that impaired patterns, as well as the severity of impairment in myocardial fatty acid imaging, may be useful in the prediction of cardiac deaths in hemodialysis patients without obstructive CAD. Particularly, focal impairment in BMIPP SPECT in the LAD area would strongly predict future cardiac death, especially acute MI death or SCD. In the multicenter cohort study B-SAFE, the significance of BMIPP SPECT in cardiac death was demonstrated in hemodialysis patients [4]. The results in this study would further strengthen the usefulness of BMIPP SPECT for risk stratification of cardiac deaths in hemodialysis patients who have potentially high risk for cardiovascular events.
